# Mild Encephalopathy/Encephalitis With Reversible Splenial Lesion Associated With Meningitis Related to Mumps Disease: An Unusual Presentation in a Male Adult

**DOI:** 10.7759/cureus.61899

**Published:** 2024-06-07

**Authors:** Aziz Ahizoune, Yassine El-Adraoui, Ahmed Bourazza

**Affiliations:** 1 Department of Neurology and Neurophysiology, Mohamed V Military Teaching Hospital, Mohamed V University, Rabat, MAR

**Keywords:** parotitis, aseptic meningitis, mers, mild encephalopathy/encephalitis with reversible splenial lesion, mumps

## Abstract

Mild encephalitis/encephalopathy with reversible splenial lesion (MERS) is characterized by mild neurological manifestations associated with spontaneously reversible lesions of the splenium of the corpus callosum. While various conditions and diseases can trigger MERS, infectious causes predominate, with mumps being notably linked to MERS in the pediatric population. Although rare in adults, there are sporadic case reports associating mumps with MERS. Here we report a 23-year-old male patient with a typical presentation of mumps who presented with meningeal syndrome, dizziness, seizures, and right orchitis. Brain MRI showed classic findings of MERS syndrome while cerebrospinal fluid analysis demonstrated lymphocytic pleocytosis. Our patient had a confirmed diagnosis of mumps disease with multiple complications, including MERS, meningitis, and orchitis, and was managed with symptomatic medications and antiviral therapy. Subsequently, there was a gradual resolution of these manifestations and the outcome was favorable, with no residual sequelae.

## Introduction

Mild encephalitis/encephalopathy with reversible splenial lesion (MERS) is a radioclinical entity corresponding to mild neurological manifestations due to damage of the splenium of the corpus callosum (SCC), which is spontaneously reversible both clinically and radiologically [1]. The main clinical features of MERS include delirium, seizures, and confusion. Radiologically, it is characterized by a reversible lesion of the SCC showing hyperintensity in fluid-attenuated inversion recovery (FLAIR) and T2 sequences with restricted diffusion, and reduced apparent diffusion coefficient (ADC) in the acute phase on brain MRI [1]. This syndrome is more frequently described in the pediatric population than in adults [2]. Several infectious agents have been reported to trigger MERS such as influenza virus, rotavirus, measles virus, adenovirus, mumps, *Mycoplasma pneumoniae*, *Streptococcus pneumoniae*, and malaria parasites [1]. MERS has also been associated with other conditions like epilepsy, withdrawal of antiseizure medications, metabolic disorders, high-altitude disease, and malignancies [1].

Mumps, also known as epidemic parotitis, is an acute viral infection that manifests primarily as parotitis. This virus is a member of the *Rubulavirus* genus of the *Paramyxoviridae *family which has a negative-sense and single-stranded RNA genome [3]. It mainly affects children during the winter and spring months, but cases have become less frequent due to widespread vaccination [4]. This acute viral infection typically presents with unilateral or bilateral swelling of the parotid glands, sometimes starting in one parotid gland and then affecting the other [3]. Furthermore, this self-limiting illness can also affect other organs such as the testes, pancreas, central nervous system, kidneys, ovaries, and heart [4]. Symptoms usually resolve without sequelae, but complications like sensorineural deafness or encephalitis may occur [4].

Cases of MERS in the context of mumps infection are rarely reported in the literature. Herein, we report MERS in a 23-year-old male patient who had manifestations of mumps associated with meningeal syndrome, and classic findings of MERS syndrome.

## Case presentation

A 23-year-old male patient with no past medical history, well vaccinated according to the national vaccination program, presented with symptoms related to a recent exposure to mumps. His 29-year-old sister had mumps 20 days ago, and a few sporadic cases were reported among close contacts. The patient was admitted to the emergency department with bilateral submandibular swelling that began 10 days ago which was followed two days later by severe headache, persistent fever, photophobia, and vomiting. Additionally, he complained of right testicular pain and dizziness. His family reported two generalized tonic-clonic seizures. Despite taking paracetamol, his fever and headache worsened.

On clinical examination, he was conscious with a fever of 39°C, with normal blood pressure and heart rate. Neurologic evaluation revealed nuchal rigidity, and positive Kernig and Brudzinski signs, with no sensorimotor deficit or dysmetria. Deep tendon reflexes and cutaneous plantar responses were normal and symmetric. Assessment of cranial nerves and cognitive function was unremarkable. Otorhinolaryngology examination by an otorhinolaryngologist, was consistent with bilateral parotid enlargement, predominantly on the right side. Further clinical examination revealed tenderness and swelling of the right testes.

**Figure 1:**
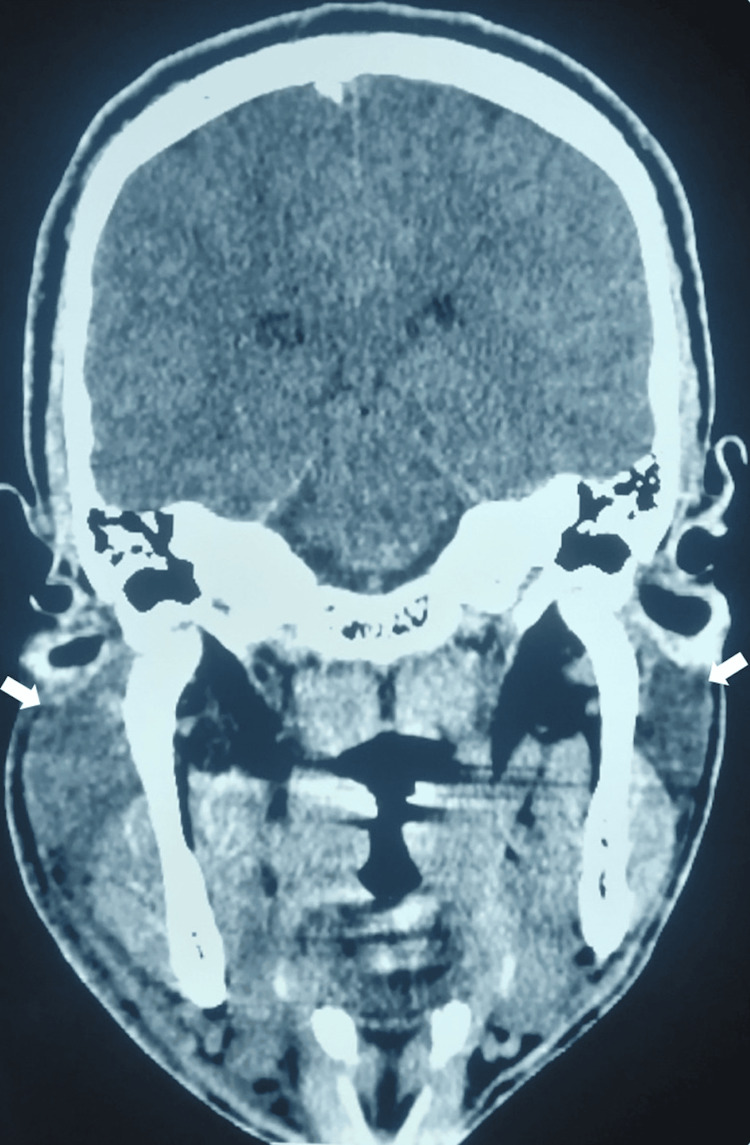
Coronal axial CT of the head demonstrating tumefaction of both parotid glands (white arrows), more prominent on the right side.

A CT scan of the brain, performed in the emergency department, showed no brain abnormalities but did reveal swelling of the parotid glands, particularly on the right side (Figure 1).

**Figure 2:**
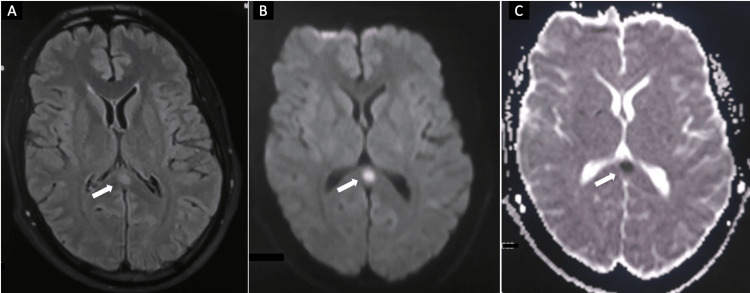
Axial brain MRI of our patient, in the acute phase, showing a lesion in the splenium of the corpus callosum demonstrating hyperintensity in FLAIR sequence (A) with high signal intensity on DWI (B), and markedly low signal intensity on ADC sequence (C). FLAIR: Fluid-attenuated inversion recovery, DWI: Diffusion-weighted imaging, ADC: Apparent diffusion coefficient.

Based on this clinical presentation, a brain MRI was conducted, revealing a lesion in the SCC with hyperintensity on T2-weighted and FLAIR sequences, diffusion restriction, and decreased ADC without contrast enhancement (Figure 2).

Given the clinical signs of meningeal syndrome, a lumbar puncture was performed, showing clear CSF with normal protein levels (Table 1). However, cytology was consistent with lymphocytic pleocytosis (121 lymphocytes/mm³ and 1 neutrophil cell). Bacterial smears and culture from CSF were negative. Further laboratory investigations revealed an elevated C-reactive protein (53 mg/l, normal value <5 mg/l). Complete blood count, renal function tests, liver function tests, and serum ionogram were normal. Mumps-specific IgM antibody test was positive. HIV serology was negative. Testicular ultrasonography showed right orchiepididymitis.

**Table 1:** Main laboratory results. CSF: Cerebrospinal fluid; RBC: Red blood cells; WBC: White blood cells.

	Results	Reference range
C-reactive protein (mg/dL)	53	(< 5 mg/L)
CSF		
Color	Colorless	
RBC (/uL)	15	(0 - 5/uL)
WBC (/uL)	122	(0 - 5 /uL)
Lymphocytes	121	-
Neutrophils	1	-
Protein (mg/dL)	26	(14 - 45 mg/dL)
Glucose (mg/dL)	50	(40 - 70 mg/dL)

**Figure 3:**
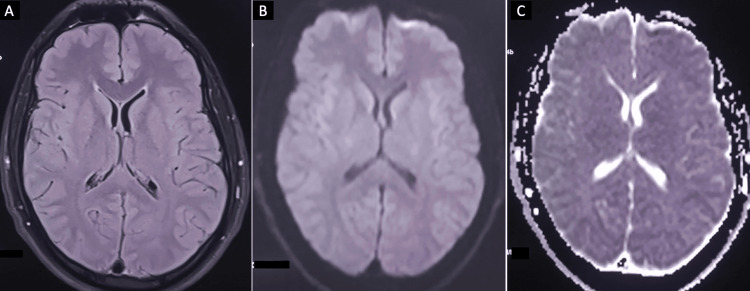
MR imaging findings on day 45, demonstrating the complete disappearance of previous abnormal intensity on FLAIR (A), DWI (B), and ADC map (C). FLAIR: Fluid-attenuated inversion recovery, DWI: Diffusion-weighted imaging, ADC: Apparent diffusion coefficient.

An electroencephalogram (EEG) was performed due to the history of seizures and showed no abnormalities. The patient was diagnosed with MERS and meningitis related to the mumps virus. Therefore, he was treated with antiviral medication and symptomatic treatment, including acyclovir (750 mg/8 hours intravenously), paracetamol, acetylleucine, and rehydration. His condition improved, with resolution of fever, headache, parotid swelling, and testicular pain. He became asymptomatic and was discharged from the hospital with no subsequent treatment after 15 days. A follow-up brain MRI on day 45 post-onset of the disease (Figure 3) showed the resolution of the previously described lesion in the SCC.

## Discussion

The typical features of MERS include the reversibility of the corpus callosum (CC) splenium lesion on brain MRI as reported in our patient, along with a moderate clinical presentation of encephalitis or encephalopathy [1]. We described here an unusual presentation of mumps in a young male adult diagnosed with meningoencephalitis, including typical findings of MERS and lymphocytic meningitis. Mumps was one of the most common viral causes of meningitis before the era of mumps vaccination [5]. Meningitis in the setting of mumps is called aseptic meningitis because the presentation is usually mild and resolves without treatment, though some cases can be severe and life-threatening [6]. In our observation, the meningeal syndrome was not apparently mild, as evidenced by the persistent fever for one week and multiple organ involvement by the viral disease. Orchitis is reported in 14% to 35% of post-pubertal males infected with mumps and is associated with epididymitis in most cases [3]. This painful inflammation of the testes is usually unilateral, with a potential risk of subsequent atrophy [3]. Our patient had unilateral orchiepididymitis which progressed favorably with treatment. 

The CC is the major commissural area of the brain and is constituted of myelinated nerve fibers that connect the two cerebral hemispheres. From front to back, the CC consists of four parts: the rostrum, the genu, the body, and the splenium [1]. The pathophysiology of the splenium lesion in MERS is not well understood, but the main causes are thought to be multiple, including intramyelinic edema, the influx of inflammatory cells and macromolecules, combined with cytotoxic edema and hypotonic hyponatremia caused by infection [1]. Cytotoxic edema is the most likely mechanism implicated, though it alone may not fully explain the reversibility of the SCC lesion [1].

The virus of mumps has already been implicated in pediatric MERS since the first description of this syndrome by Tada et al., where the term encephalitis was used for those with CSF pleocytosis and encephalopathy term for those with normal CSF [7]. In this latter study, Tada et al. reported two male children presenting with MERS who had meningoencephalitis and CSF pleocytosis secondary to mumps virus [7]. Since then, only a few case reports have been published describing pediatric MERS in the context of mumps infection, mainly in the encephalitis phenotype, and recently one case in the encephalopathy subtype [8]. Our patient was consistent with the encephalitis subtype of MERS.

Concerning MERS in adults, reports of such an association with meningitis and mumps symptoms like our case report are very exceptional. To the best of our knowledge, only two cases have been reported among adults in the literature. Firstly, Zhang et al. published a case of a 34-year-old woman who presented with dizziness, drowsiness, and fever associated with pleocytosis (7 white blood cells/mm3), normal CSF protein level, and positive mumps-specific IgM antibody [9]. Secondly, Yildirim et al. described a 26-year-old woman with apathy, attention deficit, temporal disorientation, and right parotitis with normal CSF and positive IgM serology for mumps virus [10].

Commonly, MERS is preceded by prodromal symptoms such as fever, headache, and digestive tract disturbance. Clinical manifestations of MERS are varied and non-specific. Patients generally experience; cognitive dysfunction, confusional syndrome, behavioral disturbances, epileptic seizures, somnolence, coma, dizziness, and visual disturbances [1]. Generally, symptoms of MERS disappear completely within a month. The most characteristic MRI findings are reversible hyperintense signals on T2-weighted, FLAIR, and diffusion images with a reduced signal on ADC as demonstrated in our patient. This MRI finding typically resolves on diffusion sequence within approximately two weeks [1]. We define two radiological patterns for MERS: type 1, with a small round or oval lesion isolated in the center of the SCC as showed also in our observation, and type 2, with extensive lesion of the SCC to the callosal fibers and adjacent white matter or to the anterior region of the CC [1].

MERS patients generally show full recovery, both clinically and on imaging, but some patients with the type II pattern may develop neurological sequelae [1]. Our patient had a good resolution of his multi-visceral mumps involvement. The prognosis is generally good in the setting of MERS, but some patients may experience residual sequelae, which are very rare [1].

There is no consensus in the literature on the management of these patients. Some studies show a favorable outcome, either without treatment or with the use of therapies such as methylprednisolone, intravenous immunoglobins, or antivirals [9]. In our case, we opted to use antivirals because the patient had a very prominent meningeal syndrome with persistent fever for a week. In all cases of mumps-associated MERS, including our current case, the outcome has been excellent [8].

## Conclusions

Mild encephalitis/encephalopathy with reversible splenial lesion (MERS) is characterized by reversible neurological manifestations related to transient lesion of the splenium of the corpus callosum and is more commonly reported in the pediatric population than in adults. Although various conditions can trigger MERS, infectious etiologies are predominant. The co-occurrence of MERS and meningitis is rarely reported in adult patients, with only a few case reports. Despite widespread mumps vaccination, cases of mumps continue to occur, raising questions about vaccine effectiveness in some individuals. Our observation highlights the potential for mumps to cause neurological manifestations such as MERS and meningitis, even in adult patients.
